# Les accidents de scooter chez l'enfant au CHU Aristide Le Dantec de Dakar: à propos de 74 cas

**DOI:** 10.11604/pamj.2016.23.32.8708

**Published:** 2016-02-08

**Authors:** Azhar Salim Mohamed, Gabriel Ngom, Mamadou Sow, Papa Alassane Mbaye, Souleymane Camara, Ndeye Fatou Seck, Oumar Ndour

**Affiliations:** 1 Service de Chirurgie Pédiatrique du Centre Hospitalier Universitaire (CHU) Aristide Le Dantec de Dakar, Sénégal

**Keywords:** Accident de scooter, enfant, piétons, fractures, Dakar, scooter accident, child, pedestrians, fractures, Dakar

## Abstract

**Introduction:**

Les accidents de scooter sont de plus en plus fréquents à Dakar. Le but de ce travail est de rapporter les aspects épidémiologiques et lésionnels des ces accidents chez l'enfant à Dakar.

**Méthodes:**

Une étude rétrospective et descriptive a été menée dans le Service de Chirurgie Pédiatrique du CHU Aristide Le Dantec de Dakar entre le 1^er^ janvier 2009 et le 31 décembre 2011. Nous avons étudié divers paramètres dont la fréquence, les aspects sociodémographiques et lésionnels.

**Résultats:**

Les accidents de scooter représentaient 12% des accidents de la voie publique. Ils étaient fréquents entre 12 heures et 14 heures (27%) et entre 18 heures et 20 heures (28,4%). Ils pouvaient survenir tous les jours et étaient fréquents pendant les mois d'avril (17,6%) et de juin (13,5%). Ils survenaient essentiellement dans la périphérie de Dakar (78%). La tranche de 3 à 8 ans (60,8%) était la plus touchée. Le sexe masculin était prédominant (sexe ratio de 1,5). Les piétons étaient les plus vulnérables (93,2%). La chute était le mécanisme dominant (98,7%). Les lésions touchaient surtout le membre inférieur (51,1%) et étaient constituées essentiellement de fracture.

**Conclusion:**

La fréquence des accidents de scooter chez l'enfant est liée à l'urbanisation galopante et à l'accroissement du parc automobile dans une presqu’île. Les victimes sont essentiellement des piétons et présentent le plus souvent des fractures.

## Abstract (en)

**Introduction:**

Scooter accidents are becoming more frequent in Dakar. The purpose of this study is to report the epidemiological and lesional aspects of these accidents on children in Dakar.

**Materials and methods:**

A retrospective and descriptive study was conducted in Pediatric Surgery Unit of the Aristide Le Dantec Hospital in Dakar from January 1st, 2009 to December 31, 2011. Various parameters were studied. These parameters include: frequency, sociodemographic and lesional aspects.

**Results:**

Scooter accidents represent 12% of highway accidents. They were more frequent in the day, from 12 am to 2 pm (27%) and in the night, from 6 pm to 8 pm (28.4%). They might occur every day but were more frequent during April (17.6%) and June (13.5%). They occurred mainly in the suburban district of Dakar (78%). The age group most affected was the one from 3 to 8 years (60.8%). Male gender was predominant (sex ratio1.5). Pedestrians are the most vulnerable (93.2%). The fall was the dominant mechanism (98.7%). Lesions affected mainly the lower limb (51.1%) and were essentially constituted by fracture.

**Conclusion:**

The frequency of scooter accidents in children is related to the galloping urbanization and to the increase of the number of cars on the roads in the peninsula. Victims are essentially pedestrians and present most of the time fractures.

## Introduction

L'urbanisation croissante explique les problèmes de mobilité urbaine à Dakar, la capitale du Sénégal. En effet, près du quart de la population sénégalaise habite cette ville, qui ne représente que 0,3% du territoire. D'autre part, 75% du parc automobile se trouve dans cette presqu’île; les 13 autres régions du Sénégal se partageant le reste de ce parc. Ces facteurs expliquent l'intensité du trafic routier et par conséquent l'accroissement des accidents de la voie publique. Le scooter est un moyen de transport à deux roues, assez pratique, mais pouvant être à l'origine d'accidents graves. Deux études hétérogènes, tenant compte, aussi bien des adultes et des enfants victimes de type de locomotion ont été menées à Dakar [1, 2]. Cette troisième étude rapporte les accidents de scooter survenus exclusivement chez les enfants. Le but de ce travail était de décrire la part des accidents de scooter parmi les accidents de la voie publique et de rapporter certains aspects sociodémographiques et lésionnels.

## Méthodes

L’étude a été réalisée au service de Chirurgie Pédiatrique du CHU Aristide Le Dantec de Dakar entre le 1er janvier 2009 et le 31 décembre 2011. Il s'agissait d'une étude rétrospective et descriptive. Tous les enfants victimes d'un accident de scooter et suivis régulièrement dans le dit service ont été inclus dans l’étude. La fréquence des accidents dus au scooter par rapport à l'ensemble des accidents de la voie publique a été précisée. Nous avons aussi étudié les données sociodémographiques suivantes: l’âge, le sexe, la position du traumatisé (piétons, conducteur ou passager) au moment de l'accident, l'heure (subdivisée en six tranches: 6h à 8h, 9h à 11h, 12h à 14h, 15h à 17h, 18h à 20h, 21h à 23h et 00h à 5h), le jour, le mois et le lieu de l'accident. L'accident pouvait survenir dans la zone du Plateau, qui est centre ville, qui concentre l'essentiel des affaires et des services, dans la banlieue de Dakar ou dans la périphérie de Dakar, zone comprise entre le Plateau et la banlieue. Les aspects lésionnels étudiés étaient le mécanisme, la localisation de la lésion (membres, tronc, crâne) et le type de lésions (fracture, plaie, contusion, luxations,…).

## Résultats

**Figure 1:**
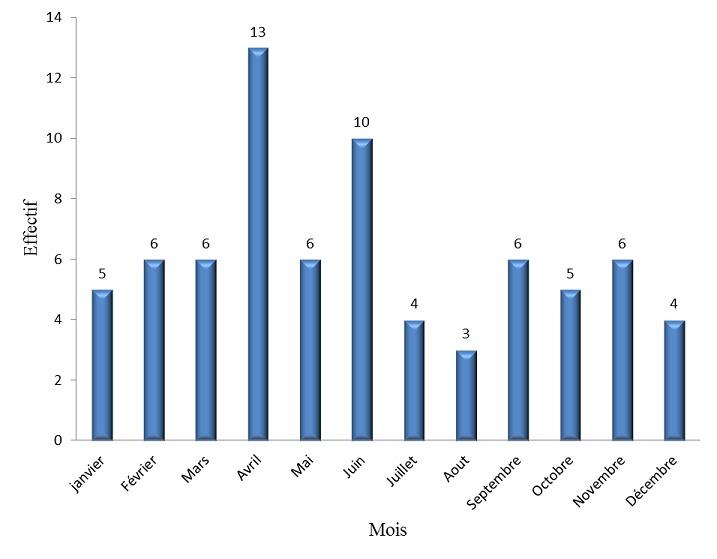
Répartition des accidents de scooter selon le mois

**Figure 2:**
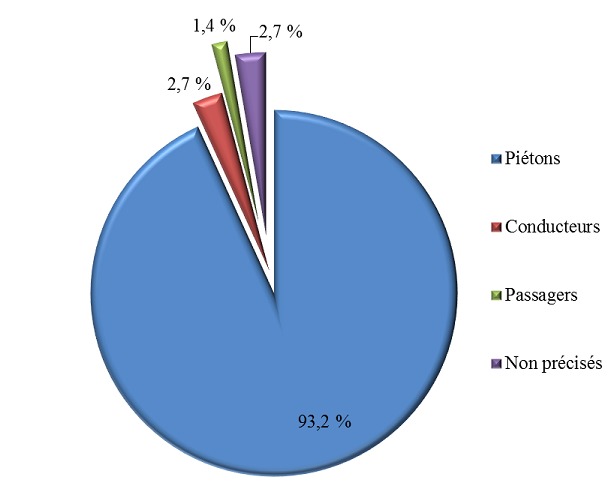
Répartition des victimes selon la position

Durant la période d’étude, nous avons répertorié 74 cas d'accidents de scooter sur un total de 615 cas d'accidents de la voie publique, ce qui représentait 12% des accidents de la voie publique. La tranche d’âge la plus touchée était comprise entre 6 et 11 ans avec 48,7% des cas, suivie des enfants âgés de moins de 5 ans (29,7%) puis des adolescents (21,6%). Il y avait une prédominance masculine avec un sexe ratio de 1,5. Les accidents de scooter étaient plus fréquents entre 12 heures et 14 heures et entre 18 heures et 20 heures (Tableau 1). Les accidents de scooter survenaient tous les jours avec un pic le lundi (Tableau 2). Il y avait deux pics de fréquence. Il s'agissait des mois d'avril et de juin (Figure 1). Les accidents de scooter étaient plus fréquents dans la zone périphérique de Dakar, soit dans 78% des cas. La banlieue et le centre ville représentaient respectivement 19% et 3% des cas. Les victimes d'accidents de scooter étaient essentiellement des piétons (Figure 2). Elles étaient heurtées par les scooters lorsqu'elles traversaient la chaussée (68,5% des piétons) ou lorsqu'elles jouaient dans la rue (31,5% des piétons). Les lésions survenaient essentiellement au décours d'une chute (98,7%). Le membre inférieur était le siège de prédilection des lésions lors d'un accident au scooter (51,1%) suivi du membre supérieur et de la tête avec 21,3% des cas chacun puis le tronc avec 6,3% des cas. Les fractures et les plaies étaient les lésions prédominantes avec respectivement 40,4% et 37,2% des cas (Tableau 3).

**Tableau 1:** Répartition des accidents selon les tranches horaires

Tranche horaire	Effectif	Pourcentage (en%)
06h – 08h	0	0
09h – 11h	5	6,7
12h – 14h	20	27
15h – 17h	8	10,8
18h – 20h	21	28,4
21h – 23h	7	9,4
00h – 05h	0	0
Non précisée	13	17,7
Total	74	100

**Tableau 2:** Répartition des accidents selon les jours de la semaine

Jour	Effectif	Pourcentage
Lundi	16	21,7
Mardi	5	6,7
Mercredi	12	16,2
Jeudi	10	13,5
Vendredi	12	16,2
Samedi	12	16,2
Dimanche	7	9,5
Total	74	100

**Tableau 3:** Répartition des lésions observées

Lésion	Effectif	Pourcentage (%)
Contusion	10	10,6
Dents	2	2,2
Fractures	38	40,4
Luxations	3	3,2
Plaies	35	37,2
Traumatismes crâniens	6	6,4
Total	94	100

## Discussion

Selon l'OMS, les accidents de scooter constituent un véritable problème de santé publique [3]. En Australie, ces accidents sont devenus plus fréquents que ceux des autres véhicules motorisés à deux roues [4]. Chez les enfants, ce phénomène, prend de plus en plus de l'ampleur. Aux Etats-Unis, les enfants de moins de 15 ans sont les plus touchés [5, 6]. Au Sénégal, une étude réalisée à l'Hôpital Général de Grand Yoff (HOGGY) à Dakar, trouvait 11% d'enfants et d'adolescent victimes d'accidents de scooter et 89% d'adultes [1]. Notre étude vient confirmer ces données avec 12% des accidents de la voie publique chez les enfants. Cette fréquence ne reflète pas exactement le nombre d'enfants victimes de ce type d'accidents à Dakar. En effet, des enfants sont pris en charge dans d'autres structures hospitalières de la capitale, au premier rang desquels le service de Neurochirurgie du Centre Hospitalier Universitaire National de Fann qui reçoit les enfants victimes de traumatismes crânio-encéphaliques. Les accidents de scooter sont plus fréquents aux heures de pointe. Ce sont des heures où le trafic routier est intense. La vitesse excessive des scooters, l'absence de respect du code de la route par les conducteurs et l'imprudence des enfants qui veulent rapidement arriver au domicile familial en constituent l'explication. Le jour n'a pas d'influence sur la survenue des accidents de scooter. Par contre ces accidents sont plus fréquents pendant les mois d'avril et de juin. Le mois d'avril correspond aux vacances de Pâques. Les enfants aménagent la rue comme une aire de jeu et sont confrontés à ces engins. Le mois de juin est une période de grande affluence. C'est la fin de l'année scolaire et la majorité des écoles organisent des fêtes. Les enfants, en allant à ces fêtes, sont confrontés dans les rues aux scooters. Le mois d'août est celui où il y a le moins d'accidents. Il coïncide avec le plein hivernage. Les enfants restent le plus souvent à la maison à cause de la pluie et le scooter n'est pas le moyen de déplacement le plus adapté.

Les accidents de scooter surviennent essentiellement dans la périphérie de Dakar. La concentration de la population et du trafic routier ainsi que l'existence de nombreuses écoles peuvent expliquer ce phénomène. La zone de Plateau est le milieu des affaires et des services, les scooters n'y roulent pratiquement pas et les écoles et leur voisinage sont sécurisés. Tout ceci explique la rareté de ces accidents dans cette zone. La tranche d’âge de 3 à 8 ans est la plus touchée par les accidents de scooter. Avant l’âge de 3 ans, l'enfant reste à la maison sous la surveillance des parents. Après cet âge, les enfants commencent à fréquenter l’école. Au Sénégal, les plus petits sont les plus vulnérables car ils ne sont pas toujours accompagnés. Ces enfants sont essentiellement des garçons car au Sénégal, ce sont eux qui jouent dans la rue, les filles étant confinées à des tâches ménagères. Il s'agit essentiellement de piétons heurtés par les scooters en essayant de traverser la chaussée. Par contre en Europe et aux Etats-Unis, les enfants victimes sont souvent des conducteurs de scooters [7, 8]. Dans notre série, les lésions surviennent au décours d'une chute et siègent préférentiellement aux membres inférieurs suivis de la tête. Ce constat est aussi fait dans des études réalisées à l'HOGGY de Dakar [1, 2] et au Mali [9]. Cela pourrait s'expliquer, d'une part, par le fait que le point d'impact du scooter se localise souvent au niveau du membre inférieur vue la hauteur de l'engin par apport à la taille de l'enfant et, d'autre part, par le fait que le manque d’équilibre expose la tête du piéton après la chute [10]. Aux Etats-Unis et en Australie, les lésions siègent aux membres supérieurs [11, 12]. Cette différence pourrait être en rapport avec le type de victimes. Dans les études que nous avons rapportées, il s'agissait essentiellement de piétons alors que dans les études américaines et australiennes, les victimes étaient surtout des conducteurs. Les fractures sont les lésions les plus fréquentes dans notre étude, suivies par ordre de fréquence décroissante des plaies, des contusions, des traumatismes cranio-encéphaliques, des luxations et des lésions dentaires. Plusieurs séries de la littérature ont montré que les fractures demeurent les lésions les plus fréquentes lors des accidents de scooter [1, 2, 11, 13, 14].

## Conclusion

Les accidents de scooter prennent de l'ampleur à Dakar du fait de son utilisation croissante. Les enfants victimes sont de petits enfants, essentiellement des piétons, qui essaient de traverser la chaussée ou qui jouent dans la rue, aux heures de pointe. Ces accidents sont plus fréquents dans la périphérie de Dakar, avec une recrudescence pendant les vacances scolaires. Les lésions surviennent essentiellement au décours d'une chute. Il s'agit le plus souvent de fractures siégeant aux membres inférieurs.

### Etat des connaissance sur le sujet

- Les accidents de scooter constituent un véritable problème de santé publique et sont devenus plus fréquents que ceux des autres véhicules motorisés à deux roues.
- Ils prennent de l'ampleur chez les enfants de moins de 15 ans vue l'usage croissant du scooter chez cette population.

### Contribution de notre étude a la connaissance

- Notre étude est une première dans notre pays.
- Les victimes dans notre contexte sont exclusivement des piétons contrairement à la littérature où sont souvent conducteurs.

Notre étude rapporte que les enfants étaient touchés essentiellement aux membres inférieurs.

## Conflits d'intérêts

Les auteurs ne déclarent aucun conflit d′intérêt.

## Contributions des auteurs

Tous les auteurs ont participé à la réalisation de l′étude ainsi qu′à la rédaction du manuscrit. Tous les auteurs ont lu et approuvé la version finale du manuscrit.

## References

[1] Soumah MM, Sy HH, Diouf AG, Sané JC (2005). Les accidents de circulation liés au scooter dans la région de Dakar. Rev Fr Dommage Corp..

[2] Bousso A, Camara EHS, Sané JC, Kassé AN, Thiam B, Sy MH (2011). Aspects épidémiologique et clinique des accidents de scooter à Dakar, Sénégal. Med Afr Noire..

[3] Organisation mondiale de la santé (OMS) Rapport mondiale sur la prévention des accidents de la circulation: Genève; 2004.

[4] Chapman S, Webber C, O'Meara M (2001). Scooter injuries in children. J Pediatr Child Health..

[5] Ashby K, Corbo M (2000). Child fall injuries: an overview. Hazard..

[6] Josefson D (2000). Scooters cause 9500 injuries in US in 8 months. BMJ..

[7] Yacoubovitch J, Lelong N, Cosquer M, Tursz A (1995). Etude épidémiologique des séquelles d'accidents à l'adolescence. Arch Pediatr..

[8] Montagna LA, Cunningham SJ, Crain EF (2004). Pediatric scooter-related injuries. Pediatr Emerg Care..

[9] Berthe K (2008). Etude épidémio-clinique des accidents de la voie publique chez les enfants de 5 à 15 ans dans le service de Chirurgie Orthopédique et Traumatologique de l'HGT (Thèse).

[10] Rutherford GW, Ingle R (2001). Unpowered scooter-related injuries: United States, 1998-2000. JAMA..

[11] Fong CP, Hood N (2004). A pediatric trauma study of scooter injuries. Emerg Med Australas.

[12] Zalavras C, Nikolopoulou G, Essin D, Manjra N, Zionts LE (2005). Pediatric fractures during skate boarding, roller skating, and scooter riding. Am J Sports Med..

[13] American Academy of Pediatric (2002). Committee on injury and poison prevention: skateboard and scooter injuries. Pediatrics.

[14] Consumer Product Safety Commission Scooter data.

